# Supplementary material for: Point-process modeling of secondary crashes
Source: PLoS One. 2023 Dec 13;18(12):e0295343. doi: 10.1371/journal.pone.0295343 (PMC10718442; doi:10.1371/journal.pone.0295343)
Supplement: S1 File — (ZIP) [file pone.0295343.s001.zip › Supplement material/Supplement document.docx]

The proposed modeling approach is demonstrated on a dataset consisting of all crashes on Interstate-Highway-4 (I-4) in Florida, USA for three years (2015 to 2017). The model is investigated in six different cities on I-4: Tampa, Plant City, Kissimmee, Orlando, Sanford, and Daytona Beach. The result obtained from the model contains stationary or constant background rate and non-stationary background rate (piecewise background rate and sinusoidal background rate) in the supplement material.

The abbreviation from the result in the dataset is explained in this document.

1. “tt” represents time (i.e., number of days from the start period).
2. “xx” and “yy” represents the latitude and longitude in a flat map scale.
3. “flag” represents the events which are with in the region of interest for analysis.
4. “bkgd” represents the background rate.
5. “prob” represents the probability of primary crash.
6. “lambd” represents the value of conditional intensity function.
7. “Secondary crash” represents the secondary crash event based on the queue time and probability values.
