# Supplementary material for: Point-process modeling of secondary crashes
Source: PLoS One. 2023 Dec 13;18(12):e0295343. doi: 10.1371/journal.pone.0295343 (PMC10718442; doi:10.1371/journal.pone.0295343)
Supplement: Attachment — Submitted filename: Reviewer comments.docx [file pone.0295343.s002.docx]

**Comments to Editor**

1. State the financial disclosure:

*We have added the following financial disclosure statement:*

*This research was funded by DOT-UTC Center for Advanced Transportation Mobility. The funders had no role in study design, data collection and analysis, decision to publish, or preparation of the manuscript.*

2. We note that Figure 2 in your submission contain [map/satellite] images which may be copyrighted. All PLOS content is published under the Creative Commons Attribution License (CC BY 4.0), which means that the manuscript, images, and Supporting Information files will be freely available online, and any third party is permitted to access, download, copy, distribute, and use these materials in any way, even commercially, with proper attribution. For these reasons, we cannot publish previously copyrighted maps or satellite images created using proprietary data, such as Google software (Google Maps, Street View, and Earth).

*Figure 2 in the manuscript features a hand-drawn representation of the Florida map. This image was crafted manually and does not fall under any copyright restrictions.*

**Reviewer(s)' Comments to Author:**

*We thank the reviewers for their comments which have helped improve the paper. Detailed responses for the comments are provided below.*

**Reviewer #1:**

1. Normally, after a primary traffic accident is reported. A nearby traffic incident is classified as the secondary traffic accident. What is the practical contribution of this study?

*The practical contribution of this study lies in its ability to identify secondary crashes from historical data, thereby providing a foundation for the development of policies and countermeasures aimed at mitigating the occurrence of secondary crashes. Most of the crash data reports do not report if a given crash is primary or secondary. By identifying and understanding the patterns of secondary crashes through weekly and daily traffic trend, rural and urban setting etc., the research contributes to the formulation of effective strategies that can effectively address the risks associated with such incidents. The results of this research including probability distribution of secondary crashes and the secondary crash average time could inform Traffic Incident Management (TIM) toward a targeted clearance time. Decreasing the time needed to clear a primary incident reduces the exposure of the scene to secondary crashes.* *We introduced a novel approach based on the self-exciting temporal point process to analyze crash events data and classify it into primary and secondary crashes.*

2. Some ground truth data should be collected to evaluate the performance of the proposed method. Some state-of-the-art methods should be considered as the benchmark models to validate the proposed method.

*The lack of available information within the crash dataset regarding the classification of crashes as primary or secondary presents a challenge in validating the model's performance. Therefore, to address this, we conducted a comparison of our outcomes from the model using a data-driven technique from the literature [1,2] as detailed in the model validation section of the original manuscript. This methodology with a data-driven approach [1,2] utilizes BlueToad® speed records to detect secondary crashes.*

*To evaluate our model's effectiveness, we applied it to analyze crash data from the I-95 highway. Our assessment focused on a 20-mile segment of the highway within two cities along the I-95 route: Jacksonville and Miami. Our model matched well with the data-driven technique in identifying the secondary crashes with 92.6% and 92.3% for Jacksonville and Miami regions respectively, demonstrating a strong alignment in effectively identifying secondary crashes in both cities.*

3. Some typos in the study should be corrected and the manuscript should be carefully proofread.

*All typographical errors in the manuscript have been rectified, and a comprehensive proofreading process has been undertaken to enhance the quality of the manuscript.*

4. With the development of the CAV technology, some traffic platooning strategies can be used to improve the traffic safety. The authors are suggested to review this field and discuss in the manuscript. For example, see: Operation analysis of freeway mixed traffic flow based on catch-up coordination platoon. Accident Analysis & Prevention. Longitudinal safety evaluation of connected vehicles’ platooning on expressways. Accident Analysis & Prevention.

*In the discussion section of our manuscript page 23-24, we included the content on connected and autonomous vehicles (CAV technology) and their role as it reduces the effect of secondary crashes.*

*The utilization of Connected and Autonomous Vehicle (CAV) technology on American freeways is currently in its initial phases. Pilot projects are aiding in the collection of data and the testing of the safety and dependability of CAV technology. As this technology advances, it is foreseeable that an increased number of CAVs will operate on US freeways. With the emergence of connected vehicle (CV) technologies, it's highly probable that CVs will soon possess the capability to communicate with each other using wireless vehicular network. This information exchange among vehicles is anticipated to alter traffic operations and empower drivers to take more proactive measures. Rahman et al. [3] conducted a study to assess the safety of CV platoons by comparing managed-lane CV platoons and all-lanes CV platoons. The study's findings demonstrated that both approaches significantly enhanced longitudinal safety on the expressway compared to the non-CV scenario. Further, Yang et al [4] suggest that the utilization of connected vehicles could serve as a practical approach to reduce the occurrence of secondary crashes. Despite projections indicating an upsurge in the adoption of connected vehicle (CV) technology in the near future, it's important to recognize that a mixed fleet of both CV-equipped and non-CV-equipped vehicles will coexist for foreseeable future. As connected vehicles are introduced into the existing traffic mix, alongside vehicles lacking CV technology, they are not exempt from the potential risks of secondary collisions despite their enhanced communication abilities.*

**Reviewer #2:** Overall, the manuscript is a well-organized paper, which proposed a new approach to identify secondary crash and evaluated with real-word dataset and compared with other existing approaches. Here are several comments/questions.

*We thank the reviewers for their comments which have helped improve the paper. Detailed responses for the comments are provided below.*

1. It seems the model was trained with 2015-2017 data to determine the parameters, it would be better to fit with the most recent data, if the data is available.

*The training process of the model, which incorporated data from 2015 to 2017, was impacted by several considerations. Notably, we began this research in mid 2021, and the clean data we had access to at that time was the 2015-2017 data. Unlike more recent data, this dataset remains unaffected by the traffic reductions caused by the fluctuations due to the COVID-19 pandemic. Because of this, we utilized this historical data for our study obtained from the Florida Department of Transportation (FDOT). We believe that this methodology can be applied to any dataset, even though historical data was employed in this instance. The study’s primary intention was to introduce a novel approach to address and classify secondary crashes. This approach relies on a self-exciting temporal point process for the analysis of crash event data, presenting a new contribution to the field of transportation research.*

2. For the model used in the manuscript, it works well with earthquake, but it may not work perfectly with crash data. The model seems only consider the time dimension of crash events, but it does not involve the spatial attribute of crash events. Instead, it assumes any event within 2 miles and obtained queue time from model is the secondary crash. The result from this approach can be quite similar with using static spatial and temporal threshold, for example 120 minutes and 2 miles. Also, when you using the 2 miles, are you only considering the crash happening within 2 miles downstream or also includes the crashes upstream?

*The self-exciting point process model deals with interconnected events that are influenced by each other. While the application of self-exciting point process models originated in the field of earthquake analysis to categorize mainshock and aftershock events from earthquake data, it has found applications across diverse domains. These domains include crime event analysis [5], tweet popularity prediction [6], epidemiological studies [7], as well as the analysis of instances involving mass killings and school shootings [8]. This is discussed in the introduction section in the manuscript. The requirement for this model involves sequential temporal data, making it a seamless fit for situations such as secondary crashes.*

*We understand that considering spatio-temporal dimensions is significant. Since this is the first time we're using this approach for secondary crashes, we wanted to introduce the concept through temporal analysis first. In the near future, we plan to include details about spatial and temporal dimensions to make the model better at predicting. But considering spatio-temporal analysis is beyond the scope of the current paper.*

*According to existing literature [9–11], a spatial threshold of 2 miles has been deemed appropriate for our present analysis, enabling the classification of any crash occurring within this radius as a secondary crash. Furthermore, our spatial scope encompasses crashes both downstream and upstream within the 2-mile radius, contributing to a comprehensive evaluation of the crash dynamics surrounding primary crash (this is added in the manuscript line 46-48).*

3. The manuscript seems only attempts to identify the crash queue time, which is at the city level, but the queue time could be quite different in different locations and sites. With the overall queue time, it may cause false positives for some secondary crashes.

*Jalayer et al. [12] found a notable disparity in the frequency of secondary crashes between urban and rural contexts. Specifically, the study highlighted a greater incidence of secondary crashes within urban settings in comparison to rural areas. Our dataset is diverse, consisting of cities of different sizes. This includes metropolitan areas like Orlando and Tampa, characterized by higher traffic densities, as well as suburban regions like Sanford and Kissimmee, where traffic densities are lower and small cities like Daytona Beach and Plant city. The queue time estimation is based on the 2-mile spatial entry and exit location points onto the freeway across various cities. This approach is inclined to capture regions with a higher frequency of crashes due to the significant traffic volume in these areas. As a result, the obtained average queue time addresses the broader freeway context.*

*However, it's important to acknowledge that queue times can vary considerably across different locations and sites within a city or along the freeway. This variability has a small chance that may lead to false positives when applying the overall average queue time to secondary crashes. Hence, we utilize region-specific queue times rather than average times.*

4. In the formulas 8 and 9, it seems the weekly trend uses the sin function, while the rush hour trend uses the cos function. Why are they different and how do you derive these formulas?

***Weekly Trend (sin Function):***

*Traffic often exhibits periodic patterns that occur over longer time intervals. The sine function is well-suited for modeling such periodic patterns due to its oscillating nature. The weekly trend in the context to crashes, there might be a certain day-to-day variation that repeats every week, such as higher accident rates during weekends and lower rates during weekdays.*

***Rush Hour Trend (cos Function):***

*For shorter time intervals, such as within a single day, one might observe patterns that have a peak during specific times, like rush hours. The cosine function is also periodic and resembles the sine function but with a phase shift of* $\frac{\pi}{2}$*. This means that while the sine function starts from its maximum, the cosine function starts from its minimum. This can be advantageous when modeling patterns that reach their peak at a certain time. For example, when analyzing data related to traffic congestion or accidents during the day, the cosine function captures the variation with higher rates during rush hours.*

*The choice of the sine and cosine functions for modeling these trends likely stems from their appropriateness for capturing periodic patterns over different time intervals. The specific formulas we have mentioned have been derived based on fitting these functions to the observed data.*

Minor suggestions:

1. Line 42-44, it would be better to update these facts with recent (2022 or 2023) statistics.

*We have updated this information in line 42-44 in the introduction section.*

*According to National Highway Traffic Safety Administration (NHTSA), motor vehicle accidents resulted in over 42,939 and 42,795 deaths in the year 2021 and 2022 respectively in the United States [13].*

2. Line 79-82, the sentences are not clear, need to be rephrased.

*We have rephrased the sentences on page 4 line 80-85 in the introduction section.*

*Recent research has assessed the influence of a primary highway crash on the occurrence of subsequent secondary crashes through the utilization of a Zero Inflated Ordered Probit (ZIOP) regression model [14]. This approach incorporates real-time traffic conditions to establish connections between the likelihood of multiple secondary crashes and various factors such as current traffic flow, geometric attributes, weather conditions, and characteristics of the initial crash following a primary collision.*

**Reviewer #3:** 1. How to define the secondary crash in this study?

*A secondary crash is a subsequent collision that occurs as a result of the aftermath of an initial primary crash within certain space and time window. In a self-excitation point process model, recent prior events increase the probability of another event happening in the near future. The conditional intensity function* $\lambda\left( t|H_{t} \right)$ *can now be expressed as,*

$\lambda(t)=\mu+A\sum_{i:t_{i}<t} \alpha.e^{-\alpha\left(t-t_{i}\right)}$ *(1)*

*Here,* $\alpha$ *indicates the decay rate in time for the occurrence of secondary crashes while A represents the amount of excitation generated by the collection of prior events. Note that* $g_{A,\alpha}$ *is the function describing the time lag* $t-t_{i}$ *between a secondary crash and corresponding primary crash. The model is function of three unknown parameters* $\theta=\left( \mu, A,\alpha\right)$*. These parameters are determined through maximum likelihood estimation while fitting the model to the crash data. Based on the functional form of the triggering function (g), the reciprocal of ‘*$\alpha$*’ gives the average time-window over which secondary crash can occur following a primary crash. For example, in the manuscript from Table 2, in case of Kissimmee* $\alpha$*^-1^ = 0.0652 days = 93.8 minutes. This implies that if a crash occurs within 93.8 minutes and a 2-mile distance from the primary crash location, the crash is related to the primary crash and can be classified as secondary crash. A detailed explanation was provided in the original manuscript in the result section under stationary background rate to identify the secondary crashes.*

2. What are the main purposes for analyzing the stationary background and non-stationary background?

*The model presented in equation (1) is founded on the assumption of a consistent and unchanging background rate, commonly referred to as the baseline. However, the crash data varies periodically on a weekly and daily basis, as shown in Fig 1 in the manuscript. To examine this variation, a non-stationary background rate is introduced. To capture the periodic traffic and crash variations through the background rate, we use two functions: (a) a step function and (b) a sinusoidal function. A step function is used to model piecewise variation in background rate. This allows the background rate to jump to different stationary levels at different time periods. Alternatively, to account for weekly and daily traffic periodicity, a sinusoidal function is used to represent the non-stationary background rate. Finally, the Akaike Information Criterion (AIC) indicates that the most suitable fit for our model involves a non-stationary background with sinusoidal function.*

3. In the discussion section, why not compare and summarize the accuracy of identifying secondary crashes from other studies?

*We have done this quantitative comparison study by comparing our methodology with a recent data-driven technique mentioned in the existing literature [1,2]. This technique involves utilizing BlueToad® speed records to identify secondary crashes through a data-driven approach. We applied this method to analyze an I-95 highway crash dataset, as was done in [2], and contrasted it with the point-process model that we developed in this research. We focused on two cities along I-95, namely Jacksonville and Miami, and examined a 20-mile segment of the highway in each city. Our model matched well with the data-driven technique in identifying the secondary crashes with 92.6% and 92.3% for Jacksonville and Miami region respectively. We obtained access to this specific dataset upon request from the authors. Unfortunately, we couldn't assess and summarize the accuracy of secondary crashes due to the unavailability of data from other studies for comparison.*

4. Some recent studies in this field should be reviewed.

*In the original manuscript we had included recent studies to identify secondary crashes in line 80-98 and to further improve the literature review we have added some of the following recent studies in identifying the secondary crashes on freeway in the introduction section page 5.*

*Zhang et al. [15] use a binary logit model to identify the contributing factors for secondary crashes on Utah highways. They develop a hierarchical ordered probit (HOPIT) model for analyzing injury patterns in primary and secondary crashes. Another recent study [16] employs machine learning techniques for forecasting the likelihood of crashes on freeways based on traffic patterns just prior to accidents, including factors like average speed and speed reductions, in combination with freeway characteristics.*

5. How to validate the accuracy of the proposed secondary crash identification approach.

*The crash data extracted from the database lacks the distinction between primary and secondary crash events, so we have compared our results with other technique that is explained in the result section under the model validation in the original manuscript. This involved contrasting our approach with BlueToad® speed records to identify secondary crashes through a data-driven approach mentioned in existing literature [1,2]. We utilized this approach to assess a crash dataset to the I-95 highway. Our analysis was centered on two cities situated along the I-95 route: Jacksonville and Miami. Within each city, we concentrated on a 20-mile stretch of the highway. The outcomes of our model closely aligned with the results obtained from the data-driven technique in effectively identifying secondary crashes within both cities.*

**References**

[1]	Salum, J. H., Sando, T., Alluri, P., and Kitali, A. “Impact of Freeway Service Patrols on Incident Clearance Duration: Case Study of Florida’s Road Rangers.” *Journal of Transportation Engineering, Part A: Systems*, Vol. 146, No. 9, 2020. https://doi.org/10.1061/jtepbs.0000411.

[2]	Salum, J. H., Kitali, A. E., Sando, T., and Alluri, P. “Evaluating the Impact of Road Rangers in Preventing Secondary Crashes.” *Accident Analysis and Prevention*, Vol. 156, No. December 2020, 2021, p. 106129. https://doi.org/10.1016/j.aap.2021.106129.

[3]	Rahman, M. S., and Abdel-Aty, M. “Longitudinal Safety Evaluation of Connected Vehicles’ Platooning on Expressways.” *Accident Analysis and Prevention*, Vol. 117, 2018, pp. 381–391. https://doi.org/10.1016/j.aap.2017.12.012.

[4]	Yang, H., Wang, Z., and Xie, K. “Impact of Connected Vehicles on Mitigating Secondary Crash Risk.” *International Journal of Transportation Science and Technology*, Vol. 6, No. 3, 2017, pp. 196–207. https://doi.org/10.1016/j.ijtst.2017.07.007.

[5]	Mohler, G. O., Short, M. B., Brantingham, P. J., Schoenberg, F. P., and Tita, G. E. “Self-Exciting Point Process Modeling of Crime.” *Journal of the American Statistical Association*, Vol. 106, No. 493, 2011, pp. 100–108.

[6]	Zhao, Q., Erdogdu, M. A., He, H. Y., Rajaraman, A., and Leskovec, J. Seismic: A Self-Exciting Point Process Model for Predicting Tweet Popularity. 2015.

[7]	Meyer, S., Fahrmeir, L., and Höhle, M. *Spatio-Temporal Infectious Disease Epidemiology Based on Point Processes*. 2009.

[8]	Towers, S., Gomez-Lievano, A., Khan, M., Mubayi, A., and Castillo-Chavez, C. “Contagion in Mass Killings and School Shootings.” *PLoS one*, Vol. 10, No. 7, 2015, p. e0117259.

[9]	Moore, J. E., Giuliano, G., and Cho, S. “Secondary Accident Rates on Los Angeles Freeways.” *Journal of Transportation Engineering*, Vol. 130, No. 3, 2004, pp. 280–285.

[10]	Hirunyanitiwattana, W., and Mattingly, S. P. *Identifying Secondary Crash Characteristics for California Highway System*. 2006.

[11]	Zhan, C., Shen, L., Hadi, M. A., and Gan, A. *Understanding the Characteristics of Secondary Crashes on Freeways*. 2008.

[12]	Jalayer, M., Baratian-Ghorghi, F., and Zhou, H. “Identifying and Characterizing Secondary Crashes on the Alabama State Highway Systems.” *Advances in Transportation Studies an international Journal Section B*, Vol. 37, 2015, p. 129. https://doi.org/10.4399/978885488868511.

[13]	NHTSA Estimates for 2022 Show Roadway Fatalities Remain Flat After Two Years of Dramatic Increases. *https://www.nhtsa.gov/press-releases/traffic-crash-death-estimates-2022#:~:text=The%20National%20Highway%20Traffic%20Safety,42%2C939%20fatalities%20reported%20for%202021.*

[14]	Xu, C., Xu, S., Wang, C., and Li, J. “Investigating the Factors Affecting Secondary Crash Frequency Caused by One Primary Crash Using Zero-Inflated Ordered Probit Regression.” *Physica A: Statistical Mechanics and its Applications*, Vol. 524, 2019, pp. 121–129. https://doi.org/10.1016/j.physa.2019.03.036.

[15]	Zhang, Z., Gong, Y., and Yang, X. “Secondary Crashes Identification and Modeling Along Highways in Utah.” *Transportation Research Record*, 2023. https://doi.org/10.1177/03611981231182394.

[16]	Zhang, Z., Nie, Q., Liu, J., Hainen, A., Islam, N., and Yang, C. “Machine Learning Based Real-Time Prediction of Freeway Crash Risk Using Crowdsourced Probe Vehicle Data.” *Journal of Intelligent Transportation Systems: Technology, Planning, and Operations*, 2022. https://doi.org/10.1080/15472450.2022.2106564.
